# Impact of Diabetes on Oncologic Outcome of Colorectal Cancer Patients: Colon vs. Rectal Cancer

**DOI:** 10.1371/journal.pone.0055196

**Published:** 2013-02-06

**Authors:** Justin Y. Jeon, Duck Hyoun Jeong, Min Geun Park, Ji-Won Lee, Sang Hui Chu, Ji-Hye Park, Mi Kyung Lee, Kaori Sato, Jennifer A. Ligibel, Jeffrey A. Meyerhardt, Nam Kyu Kim

**Affiliations:** 1 Department of Surgery, Yonsei University College of Medicine, Seoul, Korea; 2 Department of Medical Oncology, Dana Farber Cancer Institute, Harvard Medical School, Boston, Massachusetts, United States of America; 3 Sports Medicine Laboratory, Yonsei University, Seoul, Korea; 4 Department of Family Medicine, Yonsei University College of Medicine, Seoul, Korea; 5 Department of Clinical Nursing Science, Nursing Policy and Research Institute, Biobehavioral Research Center, Yonsei University College of Nursing, Seoul, Korea; University of Bari & Consorzio Mario Negri Sud, Italy

## Abstract

**Background:**

To evaluate the impact of diabetes on outcomes in colorectal cancer patients and to examine whether this association varies by the location of tumor (colon vs. rectum).

**Patients and methods:**

This study includes 4,131 stage I-III colorectal cancer patients, treated between 1995 and 2007 (12.5% diabetic, 53% colon, 47% rectal) in South Korea. Cox proportional hazards modeling was used to determine the prognostic influence of DM on survival endpoints.

**Results:**

Colorectal cancer patients with DM had significantly worse disease-free survival (DFS) [hazard ratio (HR) 1.17, 95% confidence interval (CI): 1.00–1.37] compared with patients without DM. When considering colon and rectal cancer independently, DM was significantly associated with worse overall survival (OS) (HR: 1.46, 95% CI: 1.11–1.92), DFS (HR: 1.45, 95% CI: 1.15–1.84) and recurrence-free survival (RFS) (HR: 1.32, 95% CI: 0.98–1.76) in colon cancer patients. No association for OS, DFS or RFS was observed in rectal cancer patients. There was significant interaction of location of tumor (colon vs. rectal cancer) with DM on OS (*P* = 0.009) and DFS (*P* = 0.007).

**Conclusions:**

This study suggests that DM negatively impacts survival outcomes of patients with colon cancer but not rectal cancer.

## Introduction

Colorectal cancer is the fourth most common cancer in the United States [1], fourth in men and third in women worldwide [2]. Although the incidence rate of colorectal cancer has increased rapidly worldwide during the last two decades, the incidence rate varies 10-fold among regions of the world, with the highest rates being estimated in developed countries and lowest rates in developing and underdeveloped countries [3]. Interestingly, many regions including Asia, which used to have low incidence of colorectal cancer now have significantly increased incidence of colorectal cancer. In South Korea, for example, the incidence of colorectal cancer increased significantly from 21.2 per 100,000 in 1999 to 42.1 per 100,000 in 2007 [4]. The change in lifestyle and especially increase in obesity contribute to such rapid increase in the incidence of colorectal cancer [5].

It has been well established that obesity influences the incidence of colorectal cancer [6], [7]. Obesity and associated insulin resistance are two common contributors to the development of both type 2 DM and cancer and it is not surprising to observe increased risk of colorectal cancer in type 2 diabetic patients [8]–[10]. The pathological explanation for this connection has led to a so-called hyperinsulinemia hypothesis [11]; increased insulin level could promote colorectal tumor growth and act as a cell mitogen [12]. In support of this hypothesis, positive association between serum C-peptide concentration and an increased colorectal cancer risk were found in several studies [13]–[15]. Although studies have reported a clear association of DM and hyperinsulinemia with the risk of colorectal cancer [16], association between DM and the risk of mortality in colorectal cancer patients is somewhat unclear.

Historically, colon and rectal cancers have been considered together; however, the etiology and risk factors may differ among proximal colon, distal colon and rectal cancer. Indeed, several studies [17]–[20] have reported that DM was associated with the risk of proximal colon but not with distal and rectal cancers. Until now, most studies which evaluated the association between DM and the risk of mortality either included only colon cancer patients [21]–[24] or analyzed data from colon and rectal cancer patients together [25]–[27]. Very rarely, studies report the association between DM and the risk of mortality in rectal cancer patients separate from colon cancer. This could be due to relatively lower incidence of rectal cancer than colon cancer in Western countries [28], where most studies which investigated the association between DM and the risk of mortality in colorectal cancer patients were conducted. Furthermore, to our knowledge, the risk of mortality according to the site of colon cancer (proximal vs. distal colon) with and without DM has not been studied. Only one other study reported the association between DM and oncologic outcomes in an Asian population [23], important because the impact of DM on colorectal cancer outcomes could differ by race. With growing interest in and evidence of the relationship between DM and colorectal cancer outcomes, it is important to study the effects of DM on the risk of mortality according to the specific site of cancers in the colon and rectum in Asian population. Therefore, the purpose of this study is to investigate the impact of DM on oncologic outcomes in stage I-III colorectal cancer patients and to examine whether this association varies by the site of colorectal cancer (colon vs. rectum).

## Patients and Methods

### Ethics Statement

All study procedures were reviewed and approved by the Institutional Ethics Review Board at the Shinchon Severance Hospital and conducted according to the principles expressed in the Declaration of Helsinki. Informed consent was exempted by the board due to the retrospective nature of this research.

### Study Cohort

The prospective data base used in the current study is from a single hospital (Severance Hospital, Seoul, South Korea), which included data from patients who underwent colon or rectal cancer surgery for Stage I–III disease between January 4^th^ 1995 and December 31^st^ 2007. The data base included anthropometric measurements, date and methods of surgery, size of tumor, lymph node status, family history of colorectal cancer, site of primary tumor, carcinoembryonic antigen (CEA) levels, adjuvant or neoadjuvant chemotherapy regimen, radiation therapy dose and site (if applicable), date of recurrence and date of death. Using the prospectively collected database, 4162 potential patients who underwent resection for histologically proven adenocarcinoma of the colon and rectal cancer were considered for this study. We excluded patients aged over 90 year old (N = 5) and aged less than 18 year old (N = 1). In addition, subjects whose mortality information was missing (N = 25) were excluded from the analyses. Thus, a total of 4131 subjects were included in our study analysis. The participants were followed until October 2011.

### Study Design

Patients either had DM listed in their medical history or had a DM-controlling medication listed among their medication at the time of colorectal cancer surgery were classified as diabetic. With this method, we could not distinguish whether patients had type 1 or type 2 DM and the study thus includes both type 1 and type 2 DM. However, the incidence of type 1 DM is 1.36 (95% CI 1.05–1.66) cases per year per 100,000 individuals, which is approximately 10% of that in the United States [29]. Therefore, most diabetic patients in the data base are most likely type 2 diabetics.

The primary end point of this analysis was overall survival and the secondary outcomes were disease-free survival, recurrence-free survival and colorectal cancer-specific survival. Overall survival was defined as the time from the date of surgery to death from any cause. Disease-free survival was defined as time from the date of surgery to tumor recurrence or occurrence of a new primary colorectal tumor or death from any cause. In addition, we defined recurrence-free survival as the time from the surgery to tumor recurrence or occurrence of a new primary colon tumor. For recurrence-free survival, patients who died without known tumor recurrence were censored. Colorectal specific-survival was defined as the time from the date of surgery to death from colorectal cancer-specific cause of death. In colorectal cancer-specific survival analyses, death as a result of other causes were censored. Patients were followed every three months for the first two years after surgery, every 6 months in years 2–5, then annually. Study outcomes were ascertained until October 31^st^ 2011 through linkage to the hospital data base and the National Death Registry. Patients who remained alive at the end of the follow-up period were censored.

### Statistical Analysis

The Kaplan-Meier method and log-rank test were used for overall survival, disease-free survival and colorectal cancer-specific mortality. Survival analysis assessed deaths as a result of all-causes, colorectal cancer-specific mortality as well as disease- and recurrence-free survival. Age-adjusted and multivariable-adjusted hazard ratio (HR) and 95% CIs were calculated using Cox proportional hazards models to determine the prognostic influence of DM on survival endpoints. Participants without documented and/or treated DM were used a reference group for all analyses. Covariates include age (continuous) at diagnosis, gender, body mass index (BMI) (continuous), family history of colorectal cancer in first degree relatives (Yes, No), TNM stage (I, II and III), adjuvant therapy (No adjuvant therapy, Chemotherapy only, Radiation therapy only, Chemotherapy and radiation therapy together), and the year of surgery (continuous) were extracted from medical record. Interaction was assessed using the Wald test on the cross-product of DM and other variables of interest (Age, gender, BMI, stage of disease- and site-specific effects) in a multivariate model. All *P* values were two sided. *P*<0.05 was considered statistically significant. All statistical analyses were performed using SAS 9.1 statistical software package.

## Results

### Baseline Characteristics

The mean age of the 4131 participants was 59±11.4 year old with mean BMI of 23±3.1 kg/m^2^. Out of 4131 participants, 517 participants (12.5%) had preexisting DM prior to cancer diagnosis. Compared with subjects without DM, subjects with DM were significantly older (*P*<0.001) and had higher BMI (*P*<0.001). During the follow up period, there were a total of 1037 (467 colon cancer, 570 rectal cancer) deaths, 951 recurrences (411 colon cancer, 540 rectal cancer) and 679 colorectal cancer-specific deaths (285 colon cancer, 394 rectal cancer). Baseline characteristics are summarized in table 1.

**Table 1:** Subject characteristics.

	Colon Cancer (*N* = 2183)		Rectal Cancer (*N* = 1948)	
	No DM	DM	No DM	DM
	(*N* = 1895)	(*N* = 288)	(*N* = 1719)	(*N* = 229)
Sex (Male/Female)	1091/804	181/107	1059/660	148/81
Age (year±SD)	59.1±11.6	64.0±8.8*	58.2±11.6	62.4±8.8*
≤50	378(19.9)	18 (6.3)	391 (22.7)	13 (5.7)
51 to ≤60	526 (27.8)	57 (19.8)	482 (28.0)	72 (31.4)
61 to ≤70	643 (33.9)	139 (48.3)	562 (32.7)	93 (40.6)
>70	348 (18.4)	74(25.7)	284 (16.5)	51 (22.3)
BMI (kg/m^2^ mean±SD)	23.0±3.1	23.6±3.3	23.0±3.1	23.7±3.2
<18.5	109 (5.8)	9 (3.1)	107 (6.2)	8 (3.5)
18.5 to <23	766 (40.4)	101 (35.1)	704 (41.0)	78 (34.1)
23 to <25	412 (21.7)	67 (23.3)	377 (21.9)	59 (25.8)
≥25	418 (22.1)	85 (29.5)	381 (22.2)	68 (29.7)
Missing	190 (10.0)	26 (9.0)	150 (8.7)	16 (7)
Family history (Yes %)	84 (4.4)	10 (3.5)	54 (3.1)	2 (0.9)
TNM stage				
I	302 (15.9)	47 (16.3)	468 (27.2)	59(25.8)
II	882 (46.5)	133 (46.2)	531 (30.9)	71 (31.0)
III	711 (37.5)	108 (37.5)	720 (41.9)	99 (43.2)
No positive lymph node				
0	1182 (62.4)	180(62.5)	995 (57.9)	131 (57.2)
1 to 4	554 (29.2)	83 (28.9)	495 (28.8)	77 (33.6)
≥5	156 (8.2)	25 (8.7)	225(13.1)	21 (9.2)
Missing	3 (0.2)	0	4 (0.2)	0
Preoperative CEA				
<5 ng/dl	1406(74.2)	186 (64.6)	1288(74.9)	148(64.6)
≥5 ng/dl	479 (25.3)	101 (35.1)	424(24.7)	81(35.4)
Missing	10 (0.5)	1 (.3)	7(0.4)	0
Adjuvant therapy				
No	445 (23.5)	69 (24.0)	450 (26.2)	61 (26.6)
Chemotherapy alone	1237 (65.3)	192 (66.7)	444 (25.8)	56 (24.5)
Radiation alone	4 (.2)	0 (0)	35 (2.0)	9 (3.9)
Chemo and radiation	90 (4.7)	11 (3.8)	719 (41.8)	92 (40.2)
Missing	119 (6.3)	16(5.6)	71(4.1)	11 (4.8)

SD: Standard deviation, Number (%), DM: Diabetes Mellitus, * p<0.05 Significantly different compared with subjects who did not have DM.

### Effects of DM on the Risk of Mortality Colon and Rectal Cancer Patients

**Figure 1:**
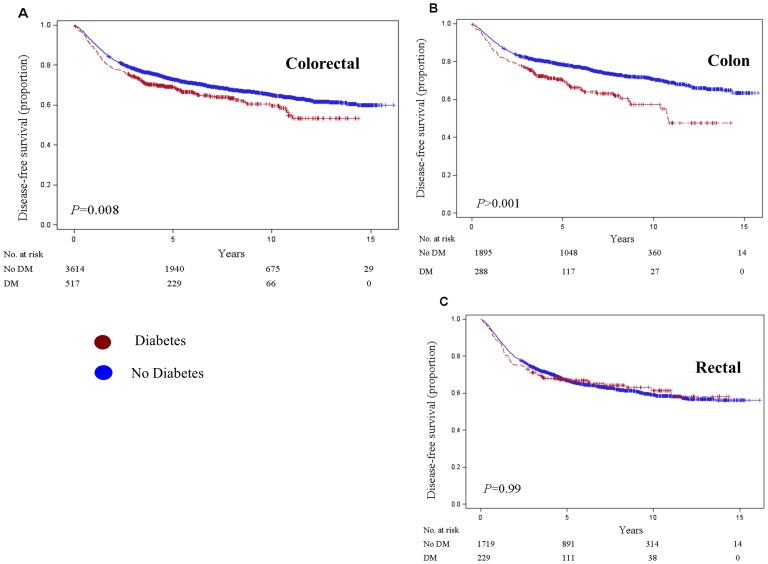
Kaplan-Meier survival curve for disease-free survival in colorectal (A), colon (B) and rectal (C) cancer patients staged I to III by diabetes mellitus status.

In Cox proportional hazard models, we examine the influence of DM on the risk of overall survival, disease-free survival, recurrence-free survival and colorectal cancer-specific survival controlling for factors associated with cancer survival (Figure 1, Table 2). There was a significant association between presence of DM and multivariate adjusted disease-free survival (HR: 1.17, 95% CI: 1.00–1.37). In contrast, there was no significant association between presence of DM and multivariate adjusted overall survival, recurrence-free survival and colorectal cancer-specific survival. To better understand the association of DM and the risk of mortality according to the site of primary tumor, we analyzed colon and rectal cancer patients separately (Figure 1). After adjustment for potential confounders, colon cancer patients with preexisting DM experienced significantly worse overall survival (HR: 1.46, 95%CI: 1.11–1.92), disease-free survival (HR: 1.45, 95% CI: 1.15–1.84) and nonsignificant trend towards worse recurrence-free survival (HR: 1.32, 95%, CI: 0.98–1.76). Preexisting DM was not associated with colon cancer-specific survival (HR: 1.25, 95% CI: 0.87–1.80). In contrast, there was no association between DM and outcome of rectal cancer patients in any of the study end points. There was significant interaction between location of primary tumor (colon vs. rectum) with DM on overall survival (*P* = 0.0094) and disease-free survival (*P* = 0.0068). No difference in the use of chemotherapy (*P* = 0.30) or radiation therapy (*P* = 0.55) between rectal cancer patients with and without DM was observed.

**Table 2:** Impact of diabetes mellitus on overall survival and disease-free survival of stage 1–3 colorectal cancer patients.

		Colorectal Cancer				Rectal Cancer				Colon Cancer			
		No. of events	No. at risk	Age, Gender-Adjusted HR (95% CI)	Multivariate HR (95% CI)	No. of events	No. at risk	Age, Gender Adjusted HR (95% CI)	Multivariate HR (95% CI)	No. of events	No. at risk	Age, Gender Adjusted HR (95% CI)	Multivariate HR (95% CI)
Overall survival													
	No DM	896	3614	1.0 (Ref)	1.0 (Ref)	508	1719	1.0 (Ref)	1.0 (Ref)	388	1895	1.0 (Ref)	1.0 (Ref)
	DM	141	517	1.09 (0.91–1.30)	1.17 (0.96–1.42)	62	229	0.89 (0.68–1.16)	0.96 (0.73–1.27)	79	288	1.35 (1.05–1.72)	1.46 (1.11–1.92)
Disease-freeSurvival													
	No DM	1127	3614	1.0 (Ref)	1.0 (Ref)	635	1719	1.0 (Ref)	1.0 (Ref)	492	1895	1.0 (Ref)	1.0 (Ref)
	DM	182	517	1.13 (0.97–1.33)	1.17 (1.00–1.37)	81	229	0.94 (0.74–1.18)	0.98 (0.76–1.25)	101	288	1.38 (1.10–1.71)	1.45 (1.15–1.84)
Recurrence FreeSurvival													
	No DM	827	3614	1.0 (Ref)	1.0 (Ref)	482	1719	1.0 (Ref)	1.0 (Ref)	345	1895	1.0 (Ref)	1.0 (Ref)
	DM	124	517	1.12 (0.93–1.35)	1.11 (0.90–1.36)	58	229	0.95 (0.72–1.25)	0.96 (0.72–1.28)	66	288	1.35 (1.04–1.77)	1.32(0.98–1.76)
Colorectal cancer-specific mortality													
	No DM	596	3614	1.0 (Ref)	1.0 (Ref)	355	1719	1.0 (Ref)	1.0 (Ref)	241	1895	1.0 (Ref)	1.0 (Ref)
	DM	83	517	1.04 (0.82–1.31)	1.08 (0.86–1.37)	39	229	0.86 (0.62–1.20)	0.92 (0.65–1.31)	44	288	1.27 (0.92–1.76)	1.25 (0.87–1.80)

HR: Hazard Ratio.

Multivariable adjustment for BMI, age, sex, family history of colorectal cancer (yes or no), TNM stage (1, 2, 3), Adjuvant therapy (No, Chemotherapy only, Radiation therapy only, Chemotherapy and radiation therapy together) and the year of surgery.

To further understand this effect by the location of colon tumor site, we further tested the association between DM and outcomes in patients with proximal and distal colon cancer. DM was associated with overall survival in patients with proximal colon cancer (HR: 2.08, 95% CI: 1.38–3.13) but not in patients with distal colon cancer (HR: 1.34, 95% CI: 0.92–1.96) (Table 3). The *P* for interaction was not statistically significant (*P* = 0.108).

**Table 3:** Impact of DM on outcome of overall survival of stage 1 to 3 colon cancer patients according to their BMI, TNM stage, age and site of cancer (proximal vs distal).

	Non DM	DM	P*_trend_*	P*_interaction_*
Risk of overall survival				
Male				
No at risk/events	1091/234	181/49		
MV adjusted (95% CI)	1.00 (Ref)	1.45 (1.02–2.06)	0.038	
Female				0.828
No at risk/events	804/154	107/30		
MV adjusted (95% CI)	1.00 (Ref)	1.48 (0.96–2.30)	0.075	
BMI specific effects of DM ?				
BMI <23 kg/m^2^				
No at risk/events	877/186	109/29		
MV adjusted (95% CI)	1.00 (Ref)	1.48 (1.06–2.05)	0.02	
BMI >23 kg/m^2^				0.672
No at risk/events	828/141	153/36		
MV adjusted (95% CI)	1.00 (Ref)	1.52 (1.04–2.21)	0.031	
Stage specific effects of DM				
TNM Stage I/II				
No at risk/events	1184/174	180/43		
MV adjusted (95% CI)	1.00 (Ref)	1.38 (1.05–1.81)	0.019	
TNM Stage III				0.369
No at risk/events	711/214	108/36		
MV adjusted (95% CI)	1.00 (Ref)	1.25 (0.84–1.85)	0.269	
Age specific effects of DM				
Age <60 years				
No at risk/events	977/145	89/20		
MV adjusted (95% CI)	1.00 (Ref)	2.04 (1.17–3.54)	0.012	
Age≥60 years				0.169
No at risk/events	918/243	199/59		
MV adjusted (95% CI)	1.00 (Ref)	1.43 (1.04–1.96)	0.026	
Site Specific effects of DM *				
Proximal Colon				
No at risk/events	798/170	128/39		
MV adjusted (95% CI)	1.00 (Ref)	2.08 (1.38–3.13)	>0.001	
Distal Colon				0.108
No at risk/events	1073/215	153/37		
MV adjusted (95% CI)	1.00 (Ref)	1.34 (0.92–1.96)	0.12	

DM: Diabetes mellitus, CI: Confidence Interval, BMI: Body mass index, MV: Multivariate, MV adjusted: Gender, BMI, TNM stage, family history of colorectal cancer (yes or no), Adjuvant therapy (No, Chemotherapy only, Radiation therapy only, Chemotherapy and radiation therapy together), the year of surgery,

? 216 case were not included due to missing BMI information,

* 31 cases were not included due to missing information on the site specific location (proximal vs distal).

### Subgroup Analysis by Gender, Age, Stage and BMI in Colon Cancer

In light of the significant association between DM and survival endpoints in colon cancer, we examined the association with DM across strata of potential predictors of survival outcomes including gender, age, BMI and TNM stage (Table 3). The association between pre-existing DM and survival endpoints in colon cancer patients was not modified by gender (*P* = 0.828), BMI (*P* = 0.672), TNM stage (stage I/II versus III, *P* = 0.369) and age (*P* = 0.169).

## Discussion

Among participants in this large cohort of Korean adults with stage I to III colorectal cancer, patients with DM experience significantly worse disease-free survival compared to non-diabetics, and nonsignificant trend towards worse recurrence-free survival and overall survival. However, when site of disease was considered, DM was associated with a significantly worse overall survival, disease-free survival and near significant recurrence-free survival (*P* = 0.065) in colon cancer patients, with no association in rectal cancer patients, suggesting that DM negatively affects the survival outcome of colon cancer patients, however, DM does not affect survival outcome of rectal cancer.

The question of the association between DM and survival outcomes in colorectal cancer patients has been previously studied, with several studies reporting a significant detrimental impact [22], [26], [30] while others have not demonstrated such associations [21], [25], [27]. Considering distinct differences in anatomy, embryology, physiology and genetics of colon and rectal cancer, studying the impact of DM on survival outcome of colon and rectal cancer patients together may not be appropriate. Most studies to date have not analyzed the relationship between DM and cancer outcomes separately in colon and rectal cancer populations with adequate subgroup sample sizes. Several prospective studies have demonstrated that DM is associated more with the risk of either colon or proximal colon cancer than rectal cancer [17], [31]–[33]. There was an only one prior study which studied the association of DM and survival outcome separately by colon and rectal cancer [34]. Van de Poll-Franse et al. [34] found significant association between preexisting DM and the risk of mortality in both colon and rectal cancer patients. It is unclear why there is discrepancy in results between the study of Van de Poll-Franse [34] and the current study. One possible explanation could include the racial differences between the two studies. Additionally, Van de Poll-Franse [34] reported that less aggressive treatment regimen was adopted for cancer patients with DM compared with patients who did not have DM. Although rectal cancer patients with DM were older than patients without DM in both studies, rectal cancer patients with DM from the study of Van de Poll-Franse et al. [34] were older than patients from the current study (71.6 vs. 62 years) and this may explain the possibility of less aggressive cancer treatment implemented in their study. Another interesting study reported that neoadjuvant chemotherapy in rectal cancer was less effective in diabetic patients than in non-diabetic patients possibly due to difference in pathologic response [35]. However, we did not observe any difference in oncologic outcome in rectal cancer patients with and without DM. In the current study, there was no difference in chemo and radiation therapy regimen between rectal cancer patients with and without DM.

In the current study, we have found stronger association between DM and survival outcome in patients with proximal colon cancer than distal colon cancer and no association between DM and survival outcome in patients with rectal cancer. It is unclear why DM was associated more with the outcomes of proximal colon cancer than with distal colon and rectal cancer. The potential mechanisms for different effects of DM on proximal, distal colon and rectal cancer could include different characteristics of tumor microsatellite instability status as well as chromosomal instability association to proximal and distal colon [20]. Considering more proximal site colon tumors (30–40%) than distal colon or rectal tumors (3–12%) have higher levels of methylated CpG islands in several tumor suppressor genes or inactivated ncRNA, worse prognosis of proximal cancer patients with DM in our study could also be associated with epigenetics which may interact between DM and tumor according to its sites [36], [37]. Furthermore, different characteristics of risk factors such as adiposity, diet and physical activity on proximal, distal colon and rectal tumor characteristics [38]–[40] may explain the different impact of DM on site-specific oncologic outcomes. The mechanism why DM was associated with colon cancer and not rectal cancer deserve more research studies since two recently reported meta-analysis studies showed that DM was associated with increased incident of both colon and rectal cancer [41]–[42]. However, this is the beyond the scope of the current study and deserve further studies.

A study of Polednak [43] raised an important question: DM could increase the all-cause mortality of colon and colorectal cancer patients, but DM may not increase the risk of colorectal cancer-specific death. In his study, he reported a 38% increased risk of all-cause mortality among colorectal cancer with DM. However, when he performed sub-analysis according to the cause of mortality, no association between DM and the risk of colorectal-specific mortality. In our study, we also found that DM was significantly associated with worse overall and disease-free survival but not with colon cancer-specific survival in colon cancer patients. Therefore, one may ask question whether DM would impact actual tumor recurrence or DM would increase risk of mortality from other causes such as cardiovascular disease. The risk of cancer recurrence was 35 percent higher in colon cancer patients with DM (HR: 1.35∶95% CI: 1.04–1.77) when age and gender were controlled. When other covariates were also controlled, the risk of recurrence was 32 percent higher in colon cancer with DM although it was not statistically significant (HR: 1.32, 95% CI: 0.98–1.76). Considering the study from Dehal et al. [44] which recently reported significantly increased cardiovascular disease-specific death in colorectal cancer patients who had DM, we may speculate that the impact of DM on mortality of colon cancer patients may be due to both recurrence of disease and death from other causes.

Although the presence of DM was not associated with oncologic outcome of rectal cancer, it was evident that the DM was associated with oncologic outcome of colon cancer [45]. Several mechanisms have been proposed to explain the link between type 2 DM and colorectal cancer including the insulin-like growth factor (IGF-1)-hyperinsulinemia theory which implies that elevated insulin and free IGF-1 levels increase the proliferation and decrease the apoptosis of colon cancer cells [46]–[47], which involves with mitogen activated protein kinases, extracellular signal regulated kinase, phosphatidylinositol-3-kinase, protein kinase B and mammalian target of rapamycin (mTOR). Another possible mechanism which links DM and colorectal cancer oncologic outcome may include altered inflammatory and anti-inflammatory cytokines in type 2 diabetic patients, which may influence the oncologic outcome of colon cancer [48]–[49].

There are limitations and strengths of the study. First, DM status was based on the past medical history and thus types of DM were not differentiated between type 1 and type 2. However, given the average age of the study participants with DM was 63 years old and the lower incidence of type 1 DM in Korea, most diabetic patients in our study would be type 2 diabetics. Furthermore, our cohort cannot address the potential of undiagnosed hyperglycemic states or DM in the control population; however, such contamination would only bias our findings towards the null hypothesis. Recent studies showed that diabetic medications and use of insulin therapy are associated with the risk and outcome or colorectal cancer patients [50]–[52]. However, the current study does not have patients’ medication as well as glycemic control data and this is the another limitation of the current study. Furthermore, the data on the use of aspirin, non-aspirin nonsteroidal anti-inflammatory drugs and cyclooxygenase-2 inhibitor in our patients was not available and therefore the use of these medications was not controlled.

In conclusion, we found significantly reduced overall and disease-free survival only in colon cancer but not in rectal patients with DM. In our knowledge, this was the first study to report the association between DM and the risk of mortality was dependent on the site of tumor (Proximal colon, distal colon and rectal cancer) in colorectal cancer.
